# Supplementary material for: Mechanisms governing the pioneering and redistribution capabilities of the non-classical pioneer PU.1
Source: Nat Commun. 2020 Jan 21;11:402. doi: 10.1038/s41467-019-13960-2 (PMC6972792; doi:10.1038/s41467-019-13960-2)
Supplement: Supplementary file 7 — Source data [file 41467_2019_13960_MOESM7_ESM.zip › Source_Data/FigureS3/FigureS3J_MotifScanOutput/homerResults/motif5.similar.html]

motif5

## Information for motif5

C
G
T
A
C
T
A
G
A
G
T
C
G
T
C
A
T
G
C
A
C
T
G
A
A
G
C
T
T
C
G
A
G
A
C
T
A
C
G
T
C
A
G
T
C
T
A
G
G
A
T
C
  
Reverse Opposite:  

C
T
A
G
G
A
T
C
G
T
C
A
T
G
C
A
C
T
G
A
A
G
C
T
T
C
G
A
G
A
C
T
A
C
G
T
C
A
G
T
T
C
A
G
G
A
T
C
G
C
A
T
  

|  |  |
| --- | --- |
| p-value: | 1e-210 |
| log p-value: | -4.842e+02 |
| Information Content per bp: | 1.641 |
| Number of Target Sequences with motif | 1730.0 |
| Percentage of Target Sequences with motif | 13.11% |
| Number of Background Sequences with motif | 1697.7 |
| Percentage of Background Sequences with motif | 4.65% |
| Average Position of motif in Targets | 378.2 +/- 307.7bp |
| Average Position of motif in Background | 321.8 +/- 192.1bp |
| Strand Bias (log2 ratio + to - strand density) | 0.0 |
| Multiplicity (# of sites on avg that occur together) | 1.07 |
| Motif File: | file (matrix) reverse opposite |

### Similar de novo motifs found

|  |  |  |  |  |  |  |  |
| --- | --- | --- | --- | --- | --- | --- | --- |
| Rank | Match Score | Redundant Motif | P-value | log P-value | % of Targets | % of Background | Motif file |
| 1 | 0.942 | G A T C G T C A G T C A C T G A A G C T C T G A A C G T A C G T A C G T C T A G G A T C G A C T C A T G G C T A | 1e-190 | -439.255706 | 13.23% | 5.03% | motif file (matrix) |
| 2 | 0.808 | T G C A G C T A G T C A A G C T T C G A A G C T A G C T G A C T T C A G G A C T | 1e-189 | -436.083699 | 28.91% | 16.65% | motif file (matrix) |
| 3 | 0.817 | C G T A A G T C G T C A G T C A C G T A A G C T C T G A A C G T G C A T C A G T T A C G | 1e-184 | -424.840489 | 22.82% | 11.96% | motif file (matrix) |
| 4 | 0.930 | A T G C G C T A T G C A G T C A A G C T C T G A G A C T G A C T C A G T C T A G G A C T G A T C | 1e-180 | -416.716674 | 8.34% | 2.28% | motif file (matrix) |
